# Corneal Biomechanics in Ectatic Diseases: Refractive Surgery Implications

**DOI:** 10.2174/1874364101711010176

**Published:** 2017-07-31

**Authors:** Renato Ambrósio, Jr, Fernando Faria Correia, Bernardo Lopes, Marcella Q. Salomão, Allan Luz, Daniel G. Dawson, Ahmed Elsheikh, Riccardo Vinciguerra, Paolo Vinciguerra, Cynthia J. Roberts

**Affiliations:** 1 Instituto de Olhos Renato Ambrósio, Rio de Janeiro, Brazil; 2 VisareRIO, Rio de Janeiro, Brazil; 3 Rio de Janeiro Corneal Tomography and Biomechanics Study Group, Rio de Janeiro, Brazil; 4 Brazilian Study Group of Artificial Intelligence and Corneal Analysis - BRAIN, Rio de Janeiro & Maceió, Brazil; 5 Department of Ophthalmology, Federal University of São Paulo, São Paulo, Brazil; 6 Life and Health Sciences Research Institute (ICVS), School of Health Sciences, University of Minho, Braga, Portugal; 7 ICVS/3B's-PT Government Associate Laboratory, Braga/Guimarães, Portugal; 8 Ophthalmology Department, Hospital de Braga, Braga, Portugal.; 9 The University of Florida Department of Ophthalmology, Gainesville, FL, USA; 10 School of Engineering, University of Liverpool – Liverpool, United Kingdom; 11 NIHR Biomedical Research Centre for Ophthalmology, Moorfields Eye Hospital NHS Foundation Trust and UCL Institute of Ophthalmology, UK; 12 Department of Surgical Sciences, Division of Ophthalmology, University of Insubria, Varese, Italy; 13 Eye Center, Humanitas Clinical and Research Center, Via Manzoni 56, Rozzano (MI) – Italy; 14 Department of Ophthalmology & Visual Science, Department of Biomedical Engineering, The Ohio State University – Columbus, OH, USA

## Abstract

**Background:**

Ectasia development occurs due to a chronic corneal biomechanical decompensation or weakness, resulting in stromal thinning and corneal protrusion. This leads to corneal steepening, increase in astigmatism, and irregularity. In corneal refractive surgery, the detection of mild forms of ectasia pre-operatively is essential to avoid post-operative progressive ectasia, which also depends on the impact of the procedure on the cornea.

**Method:**

The advent of 3D tomography is proven as a significant advancement to further characterize corneal shape beyond front surface topography, which is still relevant. While screening tests for ectasia had been limited to corneal shape (geometry) assessment, clinical biomechanical assessment has been possible since the introduction of the Ocular Response Analyzer (Reichert Ophthalmic Instruments, Buffalo, USA) in 2005 and the Corvis ST (Oculus Optikgeräte GmbH, Wetzlar, Germany) in 2010. Direct clinical biomechanical evaluation is recognized as paramount, especially in detection of mild ectatic cases and characterization of the susceptibility for ectasia progression for any cornea.

**Conclusions:**

The purpose of this review is to describe the current state of clinical evaluation of corneal biomechanics, focusing on the most recent advances of commercially available instruments and also on future developments, such as Brillouin microscopy.

## 1 INTRODUCTION

The advent of Refractive Surgery generated the fundamental need for the detection of mild forms of Ectatic Corneal diseases (ECD). As a matter of fact, these mild ectatic cases typically are at very high risk for iatrogenic progressive ectasia (keratectasia) if an additional biomechanical weakening is induced by corneal refractive procedures [1, 2] Interestingly, early detection of ectasia and monitoring its progression are also relevant due to the paradigm shift that happened on the management of ECD [3]. Corneal topography [4, 5] and corneal tomography [6] has increased our ability to identify corneal ectasia at its earlier stages, particularly prior to the patient developing symptoms, including loss in distance corrected visual acuity, or clinical slit lamp signs of ectasia [7-10]. However, despite the continued evolution of corneal shape analysis, direct clinical biomechanical measurement is recognized as paramount for augmenting the specificity and the sensitivity for identifying cases with mild disease and for characterizing the susceptibility for ectasia progression [11]. This recognition is based on a consensus that both the development of corneal ectasia and its progression are related to altered corneal biomechanics [12]. In fact, the hypothesis of ectasia progression after LASIK being related to altered biomechanical properties has been considered since the first report of ectasia [13]. Moreover, we have proposed that the enhanced screening approach for ectasia risk characterization should not be limited to the detection of mild keratoconus. Therefore, in order to define ectasia susceptibility, it is fundamental to consider data beyond classic topography and central thickness [14], including tomographic 3-D shape analysis and corneal biomechanics [11].

The current concept for ectasia development is that a focal weakening in corneal structure starts a chronic cycle of biomechanical decompensation, leading to localized thinning and steepening, which clinically define ectasia progression [15]. Interestingly, the two-hit hypothesis proposes an underlying genetic predisposition coupled with external or environmental factors, including eye rubbing and trauma [3]. In this manner, changes in curvature, elevation, and thickness occur as secondary events, leading to ocular aberrations [16], while the primary abnormality is related to a direct biomechanical weakening in the corneal stroma [15, 17].

In this review, we describe the current status of clinical biomechanical measurements of the cornea. Currently, there are two commercially available devices for *in vivo* characterization of corneal biomechanics. Newer non-commercially available research methods for clinical biomechanical assessment of the cornea will also be covered, particularly Brillouin microscopy [18].

## 2 OCULAR RESPONSE ANALYZER

**Fig. (1):**
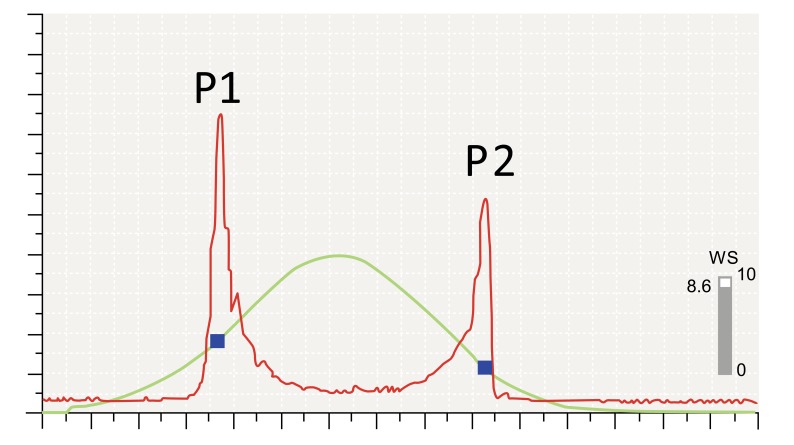
ORA Signal.

The Ocular Response Analyzer (ORA; Reichert Ophthalmic Instruments, Buffalo, NY) was introduced as the first device to evaluate *in vivo* corneal biomechanical behavior at the 2005 ESCRS meeting (Lisbon, Portugal) [19]. The ORA is a non-contact tonometer (NCT) designed to provide a more accurate measurement of intraocular pressure (IOP) through the understanding and compensation for corneal biomechanics. The ORA evaluates the response of the cornea during the bidirectional applanation process induced by an air jet pressurizing the cornea. Corneal deformation is monitored by an advanced electro-optical system that captures the infrared reflex of the corneal apex (approximately 3 mm zone) through a pinhole device [19, 20]. In this system, the maximal pressure of the air puff varies accordingly to the first applanation event. Since the maximum air puff pressure of the ORA is customized to each exam, eyes with earlier first applanation, typically with lower IOP, receive a lower maximum pressure and eyes with higher IOP receive an air puff with greater maximum pressure [20, 21]. The measurement involves auto-alignment to the corneal apex, which then triggers initiation of the air puff. The measurement takes approximately 25 milliseconds. The air pressure forces the cornea to deform (inward phase), passing first applanation when the pressure (P1) is registered. The cornea goes into a concavity configuration, until the air pressure decreases, allowing the cornea to gradually recover to its normal configuration. During the outgoing phase, it passes through a second applanation state, when the pressure (P2) is again registered. Both applanation events are recognized by the peak of the corneal reflex signal (red curve), which correspond to two independent pressure values on the air puff pressure profile (Fig. **1**). These pressure measurements (P1 and P2) are the basis for the first-generation variables reported by the original ORA software.

The term *corneal hysteresis* (CH) is a concept derived from a Greek work meaning “lagging behind” and represents the difference between these two pressures [19-21]. Corneal resistance factor (CRF) is also derived from P1 and P2,but based on the formula: P1 – kP2, where k is a constant that was developed through empirical evaluation of the relationship between P1, P2 and CCT. Actually, there is a statistically significant positive correlation between each of CH and CRF and the central corneal thickness, but the correlation is less for CH (CH, r = 0.4655; CRF, r = 0.5760) [22].

Shah was the first to report that CH had statistically significant lower values in keratoconic eyes compared to normal eyes [23]. In this early study, 207 normal eyes were compared to 93 keratoconic eyes. Eyes were diagnosed as keratoconic based on clinical examination including corneal topography. The mean hysteresis was 10.7 +/- 2.0 (SD) mmHg (range: 6.1-17.6) in normal eyes, statistically significantly higher (p<0.0001, unpaired t-test) than in keratoconic eyes with mean CH of 9.6 +/- 2.2 mm Hg (range: 4.7-16.7). Mean CCT in the normal and keratoconic eyes was 545.0 +/- 36.4 µm (range, 471-650) and 491.8 +/- 54.7 µm (range, 341-611), respectively (p<0.0001, unpaired t-test) [23]. Other reports showed that the CH and the CRF were lower in keratoconus, as well as in eyes after LASIK and surface ablation procedures [24-29]. Even though CH and CRF were found to be significantly lower in keratoconus, fundamental concepts for statistical analysis for diagnostic procedures demonstrated significant limitations [30]. There is a substantial overlap in the distributions of these parameters in normal and keratoconus cases [11, 24-27]. Fontes and coworkers compared sixty-three eyes with mild keratoconus and 80 gender- and age-matched healthy controls [27]. The mean and standard deviations for CH in keratoconic and normal controls were 8.50+/-1.36 versus 10.17+/-1.79 mmHg and for CRF 7.85+/-1.49 versus 10.13+/-2.0 mmHg for CRF. Despite the statistically significant difference of the means, the analyses of the receiver operating characteristic (ROC) curves showed a poor overall predictive accuracy for CH (cutoff: 9.64 mmHg; sensitivity: 87%; specificity; 65%; accuracy: 74.83%) and for CRF (cutoff: 9.60 mmHg; sensitivity: 90.5%; specificity: 66%; accuracy: 76.97%) regarding the detection of mild keratoconus [27].

Nevertheless, while CH and CRF have limited accuracy in detecting ectatic diseases, different approaches have been described for generating parameters from the ORA waveform signal [31-35]. The assessment related to the ORA waveform signal morphology for the diagnosis of corneal ectasia was first described on a case report of a patient who developed unilateral corneal ectasia after bilateral laser in situ keratomileusis (LASIK) [36]. In this report, the CH and CRF were almost equal in the ectatic eye and the non-ectatic eye, but there were significant between-eye differences in the ORA signals, specifically with a lower amplitude of the applanation peaks in the ectatic eye [36]. Interestingly, it was suggested to classify ORA parameters as pressure-derived parameters (CH and CRF) or corneal deformation variables; the latter of which are derived from the waveform signal [37].

The waveform-derived parameters describe corneal deformation through specific waveform features, such as the width, peak area, and height of the peaks (signal during applanation moments) [31-35]. The data from 226 normal corneas and 88 keratoconic corneas from Brazil were combined with data from Gatinel (France) and Hersh (USA) for the development of the Keratoconus Match Index (KMI), which is available on the Reichert Generation II commercial software. The KMI was developed as a single metric for detecting keratoconus and the Keratoconus Match Probability (KMP) was generated to specify the relative similarity with patterns from normality up to severe ectactic disease [38-40]. Labiris and coworkers analyzed the diagnostic accuracy of KMI and KMP in 50 keratoconus-suspect eyes compared with 50 normal eyes (control group). The mean KMI was 0.41 +/- 0.29 (SD) in the keratoconus-suspect group and 0.94 +/- 0.29 in the control group (P<.001). 27.65% of control eyes were classified as keratoconic and 10.71% of the keratoconus-suspect eyes were classified as normal, with a predictive accuracy of 94% (cut-off point: 0.72) [39].

There were also different independent groups that developed waveform parameters to characterize the temporal, applanation signal intensity, and pressure features of the corneal response [32, 33]. Dupps, Hallahan, and co-workers described that the variable with the highest overall discriminative value for keratoconus was the minimum infrared signal during corneal concavity (area under the curve: 0.985) [32]. This parameter is considered an indirect measure of maximum deformation amplitude and a more explicit indicator of corneal bending resistance than the standard ORA variables (*i.e.* CH and CRF). “Hysteresis loop area closed form”, which describes the overall viscoelastic response, was the most sensitive parameter [32]. Different groups developed distinct approaches for the interpretation of the waveform signal [33, 41].

**Fig. (2):**
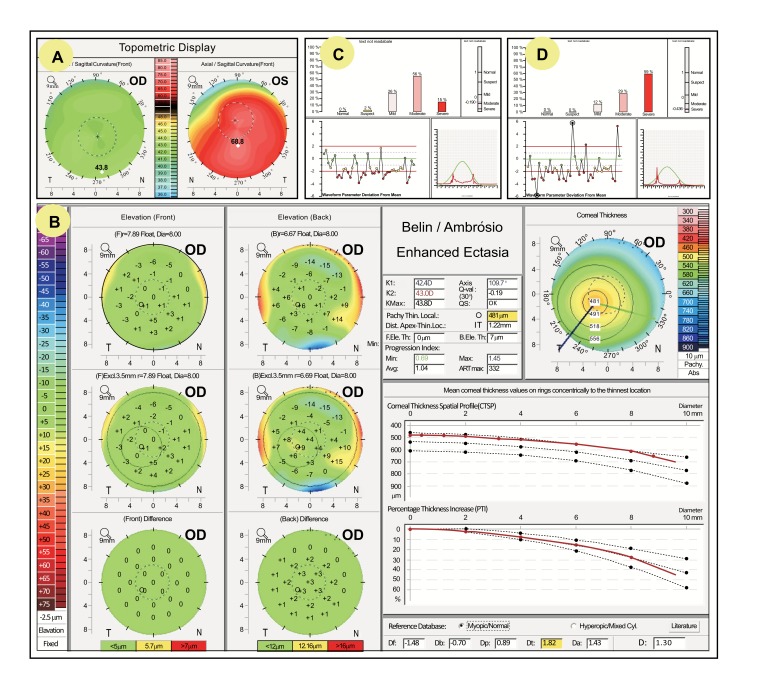
Patient with very asymmetric keratoconus. The topometric indices were within the normality in OD (A). The Belin/Ambrósio Enhanced Ectasia Display (B) demonstrated relatively normal elevation maps, but a thin cornea with 1.22 mm displacement of the thinnest point towards the inferior-temporal quadrant, an ART-Max of 322µm and a final deviation value of 1.30. The ORA KMI and KMP also allowed the detection of ectasia in both eyes (C and D).

The integration of biomechanical data with corneal tomography has been proposed for the identification of milder forms of ectasia [11, 42]. Eyes with relatively normal topography from patients with keratoconus detected in the fellow eye have been used to test and to improve algorithms to detect early disease [8, 10, 43-45]. In an example, a 29 year-old male presents with clinically advanced keratoconus in the left eye and a normal ophthalmic exam in the right eye, including distance corrected visual acuity of 20/20 (Fig. **2**). Topometric indices, derived from the front surface evaluation of Pentacam HR (Oculus; Wetzlar, Germany), are within normal limits in the right eye, including maximal keratometry of 43.8D, I/S ratio (inferior superior asymmetry at the 6 mm zone) of 0.46D (Fig. **2A**). Clinical diagnosis of keratoconus in the left eye was done according to the criteria established in the Collaborative Longitudinal Evaluation of Keratoconus (CLEK) Study [46]. The Belin/Ambrósio Enhanced Ectasia Display (Fig. **2B**) demonstrated relatively normal elevation maps in the right eye, but an abnormally thinned cornea with 1.22 mm displacement of the thinnest point towards the inferior-temporal quadrant and ART-Max of 322µm. Such pachymetric data is consistent with the diagnosis of mild keratoconus, while final deviation (D) value was 1.30, which is still on borderline, but also higher than the best cut-off for detecting FFKC [10]. The KMI and KMP derived from the ORA enabled the detection of ectasia in both eyes (Figs. **2C** and **2D**). Even though the KMI was found useful for detecting ectasia, the newest version of the ORA, the Ocular Response Analyzer® G3, does not include the keratoconus detection algorithms, focusing only on CH, which has been proven to be relevant for assessing glaucoma patients [47-50].

## 3 CORVIS ST DYNAMIC SCHEIMPFLUG ANALYZER

The Corvis ST (Oculus, Wetzlar, Germany) first introduced at the AAO 2010 meeting as a novel non-contact tonometer (NCT) system that monitors corneal deformation using an ultra-high speed (UHS) Scheimpflug camera with UV-free 455 nm blue light, covering 8.5 mm horizontally of a single slit [21, 51, 52]. The UHS system takes 4,300 frames per second, which provides true visualization and further detailing of the corneal deformation process during NCT. In contrast to the ORA, the air puff that deforms the cornea is constant in both its maximal pressure and pressure profile [20, 21, 51]. The air puff is applied concentrically on the corneal apex (first Purkinje reflex) with automatic release. Manual release is also possible.

**Fig. (3):**
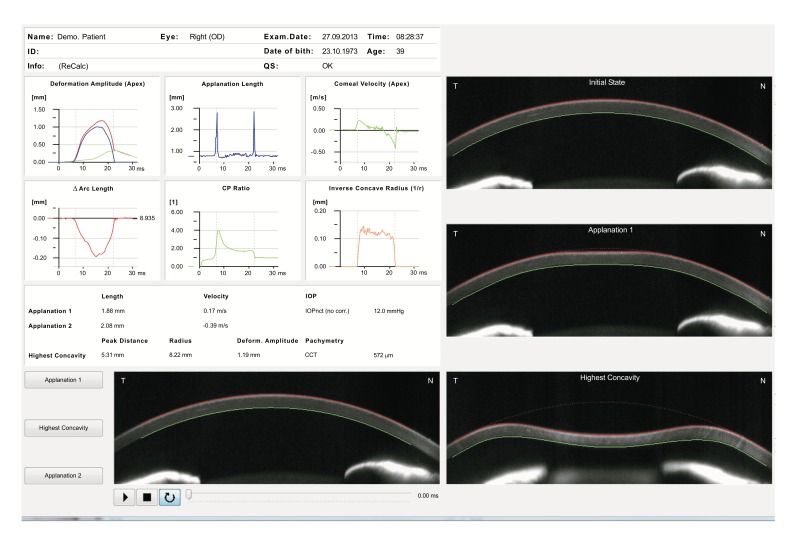
Corvis ST Overview display.

The air puff forces the cornea inwards (ingoing phase) through first applanation (inward or first applanation) into a concavity phase until it achieves the highest concavity (HC). There is an oscillation period before the outgoing or returning phase. The cornea undergoes a second applanation (outward or second applanation) before achieving its natural shape. The timing and corresponding pressure of the air puff are monitored during the complete measurement so that the correlation of the corneal state and the air pressure are identified [52]. The Corvis ST provides a set of corneal deformation parameters based on the dynamic inspection of the corneal response during the NCT process (Fig. **3** and Table **1**) [20, 21, 51]. The camera starts capturing images marking the zero value for the time a few milliseconds before the initiation of an air pulse from the pump. From the images taken prior to corneal motion, thickness and curvature data are obtained. Corneal thickness is measured by the horizontal Scheimpflug image. This allows for the calculation of the rate of increase of corneal thickness from the apex towards nasal and temporal sides. The characterization of the thickness profile enables the calculation of the Ambrósio Relational Thickness through the horizontal meridian (ARTh) [53], which is a relative simplification of the tomographic relational thickness calculations available on the Pentacam [54].

**Fig. (4):**
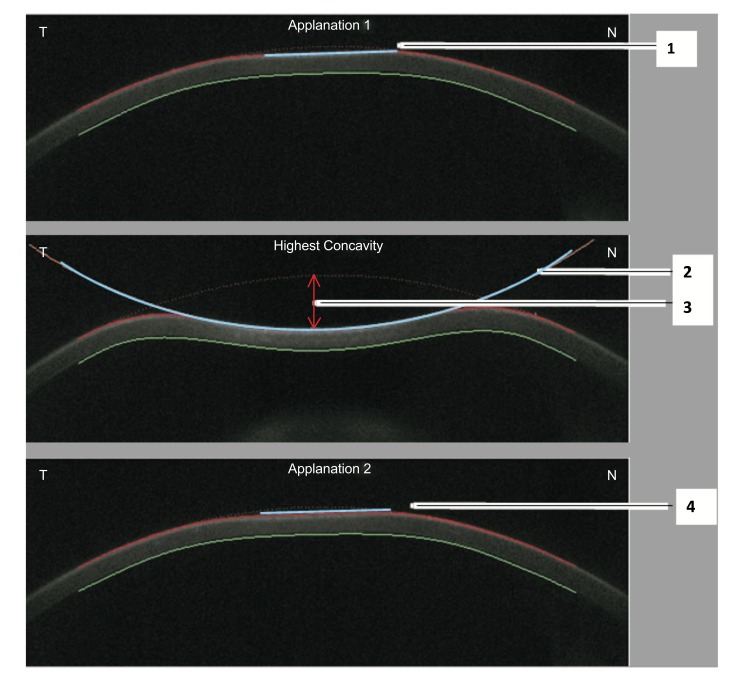
Scheimpflug images representing the ingoing applanation (above), highest concavity (middle), and outgoing applanation (below) moments. Numbers 1 and 4 are the applanation lengths at the ingoing and outgoing phases, respectively. Number 2 represents the radius of curvature at highest concavity or inverse concave radius. Number 3 represents the deformation amplitude at the highest concavity moment.

As mentioned previously with the ORA, the cornea deforms inwards due to the influence of the air pulse. The applanation of the cornea is defined by the transition from a convex to a concave shape in a zone 0.5 mm in diameter around the corneal apex [55]. The time of first applanation can be determined with high accuracy by the interpolation between single frames, with applanation time reported to 0.001ms. This interpolated time is correlated to the pressure value of the air pulse that is measured within the nozzle. A calibration factor is used to calculate the IOP value based on the applanation pressure that best matches the standard Goldmann applantion tonometry [52]. A finite element method was applied and clinically-validated for the correction of IOP (IOP FEM) based on data beyond central corneal thickness (CCT) and age [56], including corneal deformation response [57-59]. The applanation length is the line that describes the applanated part of the cornea, defined as having a constant slope (Fig. **4**). The same measurement is applied for the second corneal applanation moment that occurs during the outgoing phase. Corneal velocity is registered at the corneal apex through the measurement and recorded at both applanation times, the device also determines the velocity [52, 55].

**Fig. (5):**
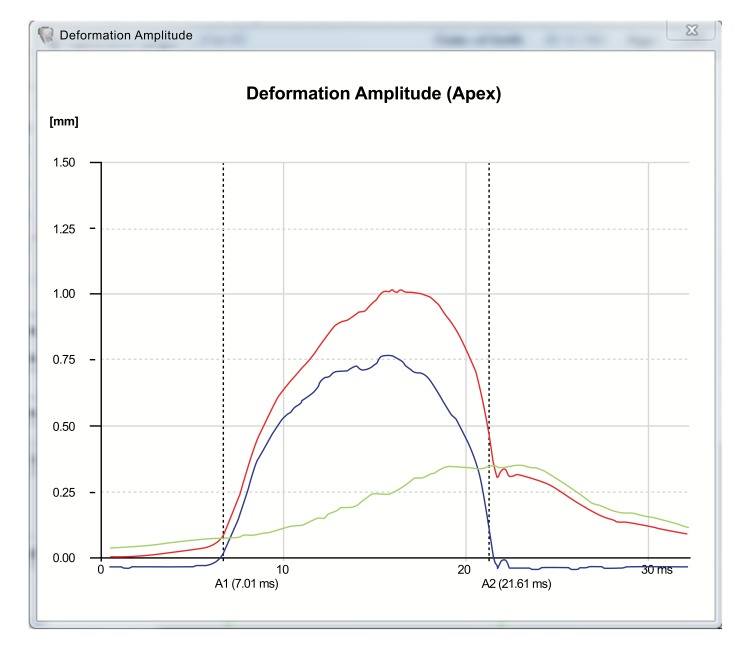
Deformation amplitude, deflection amplitude and whole eye movement parameters graphic representation (plotted versus the time). The deformation amplitude is the sum of deflection amplitude and whole eye movement.

**Fig. (6):**
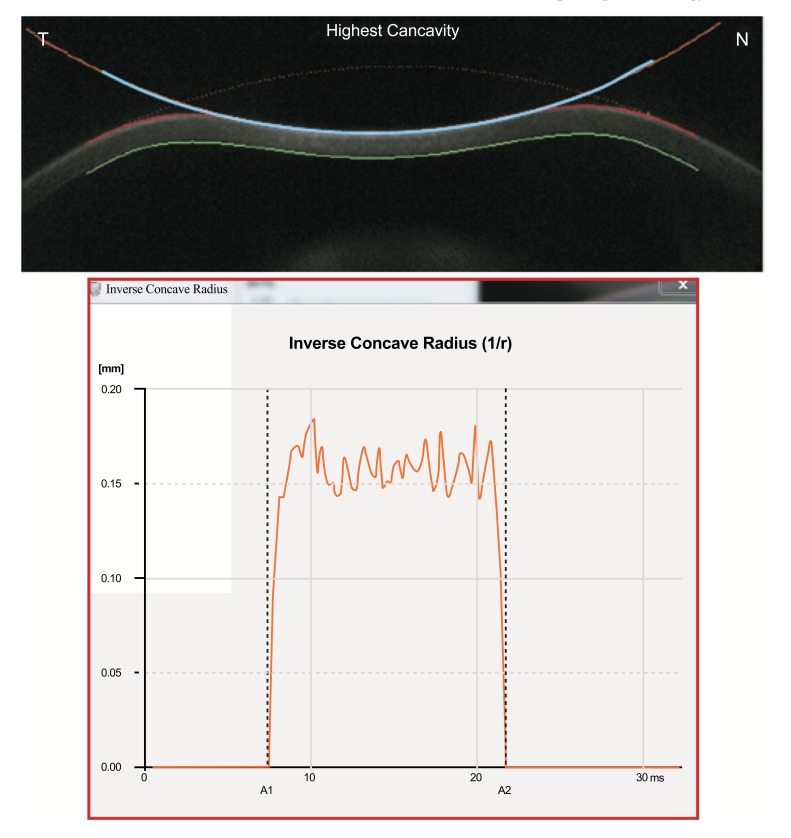
Radius of curvature at highest concavity or inverse concave radius algorithm (above). The parameters has also a graphic representation that is plotted versus the time (below).

The Corvis ST provides a set of corneal deformation parameters including analysis of those that occur at the highest concavity moment [20, 52, 55]. The deformation amplitude refers to the movement of the corneal apex in the anterior-posterior direction and is determined as the highest displacement of the apex at the highest concavity moment (Fig. **5**) [20, 52, 55]. During the measurement, there is a slight, but significant movement of the whole eye. As the cornea deforms and approaches maximum displacement, the whole eye displays a slow linear motion in the anterior-posterior direction. Typically, when the cornea reaches maximum displacement, the whole eye motion becomes more pronounced and nonlinear in nature, as the air puff pressure continues to increase to a consistent maximum. The deformation amplitude is indeed the sum of actual corneal deflection amplitude and the whole eye movement (Fig. **6**). The nasal and temporal edge points that are 4 mm-away from the corneal apex are used to track the whole eye movement, which can be seen in the video of corneal deformation, especially near the end of the air puff where the corneal deflection has already recovered [55, 60].

**Fig. (7):**
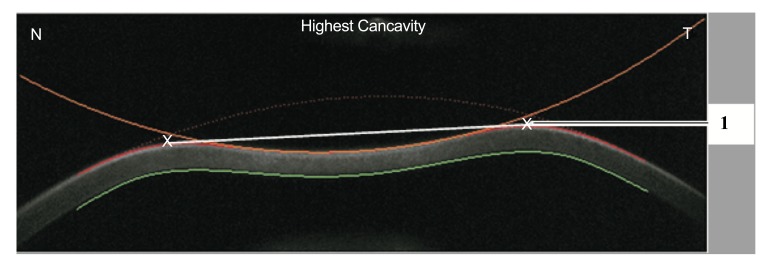
Demonstrative scheme of the peak distance parameter. It describes the distance between the two highest points of the cornea at the highest concavity moment (1).

**Fig. (8):**
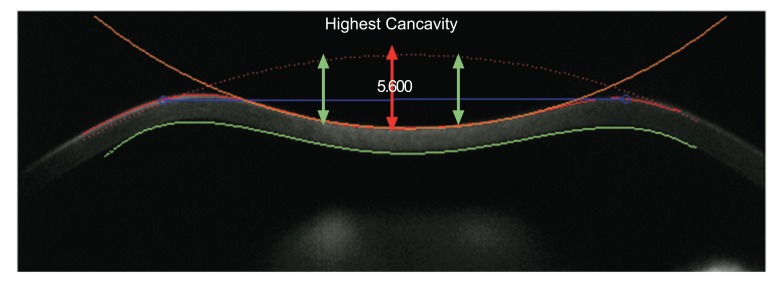
Demonstrative scheme of the deformation amplitude ratio between the apex and at 2 mm from the apex (DA Ratio 2 mm). It describes the ratio between the deformation amplitude at the apex (red arrow) and the average deformation amplitude at a 2 mm nasal and temporal zone (greens arrows).

**Fig. (9):**
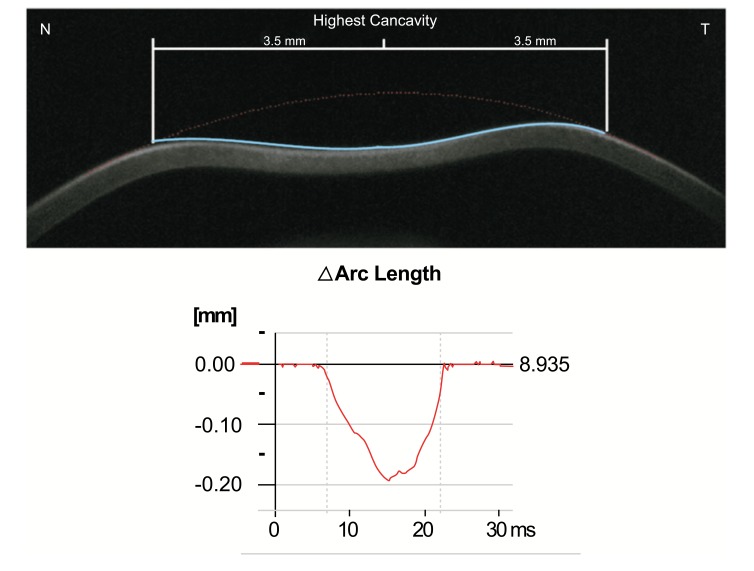
Demonstrative scheme of the delta arc length. It represents the change of the arc length during the highest concavity moment in a defined 7 mm zone.

In addition, the radius of curvature at highest concavity or inverse concave radius is calculated based on a parabolic fit and it is plotted versus time (Figs. **3** and **6**) [20, 52, 55]. The peak distance describes the distance between the two highest points of the cornea’s temporal-nasal cross-section at the highest concavity moment (Fig. **7**) and this is not the same as the deflection length [20, 55]. A parameter designated by deformation amplitude ratio is calculated as the ration between the deformation of the apex and the average deformation amplitude 2 mm from the apex (DA Ratio 2 mm; (Fig. **8**) [55]. It is expected to have a weak correlation with IOP [55]. The delta arc-length describes the change of the arc-length during the highest concavity moment from the initial state, in a defined 7 mm zone. This parameter is calculated 3.5 mm from the apex to both sides in the horizontal direction (Fig. **9**) [55]. The temporal changes in the delta arc-length are also calculated.

The relevance of the dynamic Scheimpflug evaluation as a tonometer was summarized in a film produced by Ramos and coworkers (Scheimpflug Revelations, available at https://www.youtube.com/watch?v=VQj1pVexW8c) for different clinical situations. Faria-Correia and coworkers described that ocular hypertension in pressure-induced stromal keratopathy was associated with lower deformation response along with steepening and thickening of the cornea [61]. Valbon and coworkers described the relationship between the corneal biomechanical response with age in healthy eyes [62]. In this study, the highest concavity time (the time from starting until the highest concavity is reached) correlated significantly with age [62].

The utility of the Corvis ST for ectasia detection has been studied since its prototype device [11, 63]. In a retrospective study [63], one eye per patient was enrolled in four groups based on clinical data, including Placido disk-based corneal topography and Pentacam HR (Oculus, Wetzlar, Germany) corneal tomography. Group N was comprised of 177 normal eyes, randomly selected from 177 patients, group KC was comprised of 79 eyes randomly selected from 79 patients with clinical keratoconus detected in both eyes. Group FFKC was comprised of 20 eyes with normal topographic patterns from patients with clinical keratoconus in the fellow eye, and group Stable-KCS was comprised of 16 eyes with highest topographic abnormality from 16 patients with keratoconus suspect-patterns, but with documented (>1year) stability and normal tomography. The hypothesis of this study was that the groups N and Stable-KCS would have different deformation responses compared to ectatic corneas (FFKC and KC groups). The first and second applanation times and lengths were registered, along with other metrics that were computed in the first generation software of the instrument. Considering the N and KC groups, statistically significant distributions were found for all studied parameters (Mann-Whitney, p<0.05). However, there was a significant overlap and the best parameter was the radius of curvature at highest concavity (area under the curve: 0.852). The BrAIN group (Brazilian Study Group of Artificial Intelligence and Corneal Analysis) created a linear regression analysis (LRA) model for the combination of parameters in order to maximize the separation between N and KC groups. This model was designated by Corvis Prototye-Factor (CPF-1) and presented an AUC of 0.945. Interestingly, there were significant differences among the groups (Kruskall-Wallis Test, p<0.001). The post hoc Dunn's test found no differences on CPF-1 for the FFKC and KC groups and for the N and KCS groups, but there were significant differences for N vs FFKC, N vs KC, stable-KCS vs FFKC and stable-KCS vs KC [63].

Other studies that focus on keratoconus diagnosis with the Corvis ST device are also available in the literature [64-70]. A comparative study enrolled 52 keratoconic eyes and 52 normal eyes to compare the corneal deformation response parameters between the groups [71]. In this study, the majority of the biomechanical variables (deformation amplitude, maximum corneal inward velocity, maximum corneal outward velocity, and maximum deformation area) were significantly different between the groups. In the ROC curve analysis, the maximum corneal inward velocity was the best predictive parameter with an area under the curve of 0.79 [71]. Another study described that the deformation amplitude parameter was the best predictive parameter (area under the curve of 0.882), but there was a significant overlap between keratoconic and normal corneas [66].

**Fig. (10):**
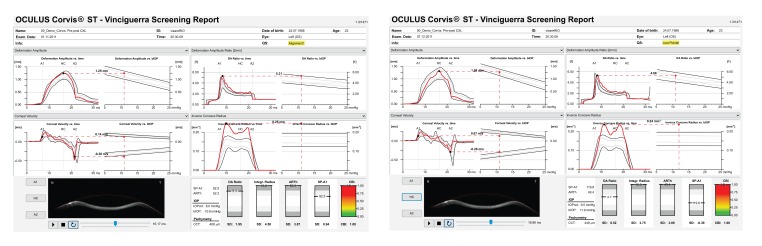
Vinciguerra Report Before (A) and After (B) Crosslinking.

A multicenter international task force group for studying the Corvis ST was initiated in 2014 involving the Eye Center, Humanitas Clinical and Research Center (Milano, Italy), the Rio de Janeiro Corneal Tomography and Study Group at the Instituto de Olhos Renato Ambrósio (Rio de Janeiro, Brazil), and The Ohio State University (Columbus, USA). The first result of such collaborative effort was to develop a comprehensive display for facilitating the understanding of corneal deformation parameters considering intraocular pressure, which also includes an equation for intraocular pressure (IOP) correction, reducing reliance of IOP measurements on both corneal thickness and age [59]. The Vinciguerra display provides a good resource for evaluating the effect after corneal crosslinking. For example, a 23 years old had the Cretan protocol [72] with UVA radiance of 18mW/cm^2^ for 5 minutes for delivering a dose of 5.4mJ, for treating progressive ectasia. The Corvis ST findings illustrate the effect of crosslinking with significant evidence of stiffening as illustrated in Figure 10, with the increase in SP-A1 [89] (Stiffness Parameter at first applanation) from 92.5 to 119.8 and decrease in DA Ration from 5.3 to 4.7 Fig. (**10**). In addition, a corneal biomechanical index (CBI) was calculated based on logistic regression analysis (LRA) to maximize accuracy in detecting keratoconus. As described by Vinciguerra and coworkers [73], the Corvis Biomechanical Index (CBI) was developed using linear regression analysis (LRA) for combining parameters from the deformation corneal response (DCR) and from the horizontal thickness profile [53], leading to high accuracy to detect clinical keratoconus [73]. 180 keratoconic patients and from 478 normals, the CBI had an AUC of 0.977, with 97.5% specificity and 94.3% sensitivity [73]. While other research studies proved the usefulness of dynamic Scheimpflug data for ectasia diagnosis [64-67, 71, 74-80], this is promising that the Corvis ST can be combined with other diagnostic instruments such as the Pentacam (Oculus Optikgeräte GmbH, Wetzlar, Germany). A novel software was developed for the integration of the Corvis ST and Pentacam, demonstrating the added benefit of corneal deformation data to the geometric analysis for the diagnosis of very mild forms of ectasia (Ambrósio and coworkers, Poster ESCRS 2015). The TBI (Tomographic/Biomechanical Index) was also introduced as a novel parameter based on a robust and innovative combination of data derived from Scheimpflug based corneal tomographic and biomechanical analysis from Pentacam HR and Corvis ST exams. The TBI resulted in very high accuracy for detecting ectatic corneal diseases (ECD), with a virtually perfect separation of normal and eyes with frank ectasia. The TBI accuracy cut off all previously analyzed parameters, which is really appreciated when analyzing cases with normal topography from patients with very asymmetric presentation with clinical ectasia in only one eye, which we found over 90% sensitivity and less than 5% of false positives (*Ambrósio et al*. Integration of Scheimpflug-based Corneal Tomography and Biomechanical Assessments for Enhancing Ectasia Detection JRS 2017, in press).

## 4 CLINICAL EXAMPLE: FORME FRUSTE KERATOCONUS

**Fig. (11):**
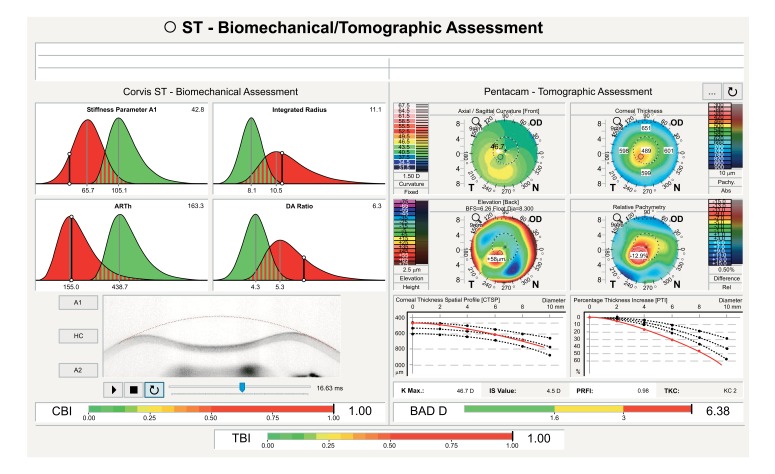
Ambrósio, Roberts & Vinciguerra (ARV) Biomechanics and Tomographic Assessments with TBI of the Right Eye with clinical ectasia from a patient with highly asymmetric ectasia, with left eye presented in Fig. **12**.

**Fig. (12):**
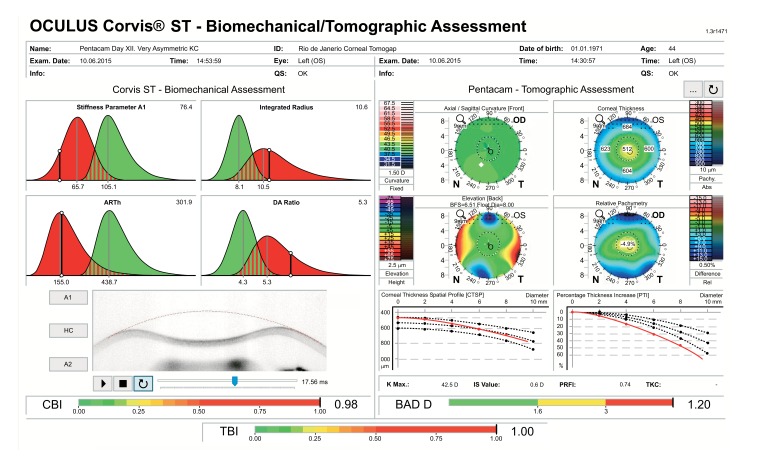
Ambrósio, Roberts & Vinciguerra (ARV) Biomechanics and Tomographic Assessments with TBI of the Left Eye with normal topography.

A 45 years-old man with very asymmetric ectatic corneal disease is presented in the Figs. (**11** and **12**). The classic “crab-claw” pattern was noted in the right eye. Left eye had a relatively normal pattern with a very mild asymmetry. Classic inferior-superior (IS-value) value at 6 mm (3 mm in diameter) [81] was 4.1 D in the right eye e and 0.6D in the left eye. Curvature maps derived from Placido-disk based topography were similar to the ones derived from Scheimpflug tomography. Despite presenting a relatively normal anterior curvature map, the left eye revealed abnormal findings in the pachymetric progression data (Fig. **11**), including an “inferior escape” from the normal mean on the Percentage of Thickness Increase (PTI) graph, and an ARTMax (Ambrósio Relational Thickness to the maximal pachymetric progression meridian) value of 304 microns, which is lower than 344 microns – the best cut off for detecting keratoconus (95.78% sensitivity and 98.5% specificity), [82]. The BAD-D was 1.2 which is borderline. [10] The CBI from CorVis ST was 1.02 in the right eye and 0.82 in the left eye, which are higher than the best cut off of 0.47 and thereby consistent with corneal ectasia diagnosis for both eyes (Fig. **12**). Considering corneal tomography and biomechanics, the diagnosis of forme fruste keratoconus was concluded for the left eye. Similar cases with normal tomography and abnormal CBI were described by Vinciguerra.

## 5 OTHER METHODS FOR IN VIVO BIOMECHANICAL ASSESSMENT

Other approaches that combine deformation of the cornea with analysis of high-speed imaging have been proposed, such as swept-source OCT or supersonic shear-wave imaging technology [15, 17, 20]. However, these techniques are not available commercially for clinical use. The Brillouin optical microscopy is another technology that has been proposed to measure *in vivo* corneal biomechanics through the analysis of light scatter [83, 84]. The interaction of photons of incident light with the acoustic phonons in the corneal tissue results in scattering. A *phonon* is the unit of vibration of the lattice structure that makes up a material. In this concept, photons gain or lose energy from interaction with phonons, and this change (gain or loss) corresponds with a shift in frequency in the Brillouin spectrum of the scattered light [83, 84]. This change is related to the elastic modulus (M ´) of the material, as shown in this equation (ρ = mass density, λ = wavelength, Ω and n = the refractive index): M'=ρλ^2^Ω4/^2^n^2^.

This technology led to new insights regarding corneal biomechanics in ectatic diseases. Brillouin imaging showed differences between healthy and keratoconic corneas [85]. Interestingly, it is revealed that the mechanical weakening is primarily concentrated within the area of the corneal protrusion [85]. Outside the diseased area, the Brillouin shift was comparable with that of healthy corneas [85]. Recently, this technique was also applied in laboratory studies to evaluate the effect of corneal CXL. Scarcelli and coworkers used Brillouin imaging to evaluate the frequency shift in porcine corneas following CXL according to several different protocols, such as epithelium-off and epithelium-on modalities [86]. This technology has been revealed to be an interesting tool to quantify the mechanical changes induced by the procedure. In this study, Brillouin corneal stiffness increased significantly (P < 0.001) by both modalities of CXL. The technique was also sensitive to identify differences in the amount of stiffening from anterior to posterior part of the cornea and to detect differences between the CXL protocols [86].

Despite new insights associated with Brillouin microscopy, there are some drawbacks and challenges that limit its implementation as a clinical tool. The intensity and frequency shifts of the scattered Brillouin light are small. Due to this reason, the device needs the use of a single-frequency laser, with large collection efficiency confocal microscopy optics, and a spectrophotometer with an ultrasensitive detector. These design features make the imaging system sensitive to temperature, vibration, and alignment. Although these factors are controlled in a laboratory setting, the transition into an accurate and reproducible commercially available clinical tool is a hurdle that has yet to be overcome.

## CONCLUSION

Direct evaluation of *in vivo* corneal biomechanics promises to provide the best analysis available for understanding corneal behavior so that detection of ectatic diseases and characterization of ectasia susceptibility can be possible. [17, 87]. Featuring the absence of relevant signs in topo and tomography scans, an initial pre-clinical phase of an ectatic disorder could be, theoretically, first detected by biomechanical measurement. In addition to safety, such evaluation may also allow for the customization of treatments for refractive and therapeutic procedures [15]. The integration of corneal tomography and biomechanical assessments with finite element model studies is promising.

Although different studies have tried to evaluate biomechanical response, primarily using the dynamic bidirectional applanation devices, it is difficult to obtain precise conclusions. The interpretation of biomechanical parameters is challenging because of the complexity of the corneal viscoelastic behavior, including the area of cornea being measured biomechanically with the two commercially available air puff devices relative to the focal area of chronic biomechanical failure that is occurring, and the impact from intraocular pressure (*i.e.*, higher intraocular pressures correlate with stiffer corneas). However, this is a very active area of research and developments can occur quite rapidly. Different approaches for interpretation of the data from these devices may prove clinically useful. In addition, other technologies for direct biomechanical assessment are also currently under development and seem promising, especially Brillouin microscopy [85-91].

## Figures and Tables

**Table 1:** Corneal deformation parameters provided by the Corvis ST.

CORVIS ST - PARAMETERS	
1st Applanation	Moment at the first applanation of the cornea during the air puff (in milliseconds). In parenthesis is the length of the applanation at this moment (in millimeters).
Highest Concavity	Moment that the cornea assumes its maximum concavity during the air puff (in milliseconds). In parenthesis is the length of the distance between the two peaks of the cornea at this moment (in millimeters).
2nd Applanation	Moment at the second applanation of the cornea during the air puff (in milliseconds). In parenthesis is the lenght of the applanation at this moment (in millimeters).
Maximum Deformation	Measurement (in millimeters) of the maximum cornea deformation during the air puff.
Wing Distance	Length of the distance between the two peaks of the cornea at this moment (in millimeters)
Maximum Velocity (in)	Maximum velocity during the ingoing phase (in meters per seconds [m/s])
Maximum Velocity (out)	Maximum velocity during the outgoing phase (in meters per seconds [m/s])
Curvature Radius Normal	Radius of curvature of the cornea in its natural state (in millimeters)
Curvature Radius HC	Radius of curvature of the cornea at the time of maximum concavity during the air puff (in millimeters)
Cornea Thickness	Measurement of the corneal thickness (in millimeters)
IOP	Measurement of the intraocular pressure (in millimeters of Mercury [mmHg])
